# ASSOCIATION OF PRO-INFLAMMATORY DIET WITH FRAILTY ONSET AMONG ADULTS WITH AND WITHOUT DEPRESSIVE SYMPTOMS

**DOI:** 10.1093/geroni/igae098.0654

**Published:** 2024-12-31

**Authors:** Courtney Millar, Alyssa Dufour, James Hebert, Nitin Shivappa, Olivia Okereke, Douglas Kiel, Marian Hannan, Shivani Sahni

**Affiliations:** Hebrew SeniorLife, Roslindale, Massachusetts, United States; Hebrew SeniorLife, Boston, Massachusetts, United States; University of South Carolina, Columbia, South Carolina, United States; University of South Carolina, Columbia, South Carolina, United States; Massachusetts General Hospital, Boston, Massachusetts, United States; Harvard Medical School, Boston, Massachusetts, United States; Harvard Medical School, Boston, Massachusetts, United States; Hebrew SeniorLife, Boston, Massachusetts, United States

## Abstract

Dietary inflammation is associated with increased risk of frailty. Those with depressive symptoms may be at higher risk of frailty onset since they typically have higher levels of inflammation. The study objective was to determine the association between a pro-inflammatory diet and frailty onset in those with and without clinically relevant depressive symptoms. This prospective study included 1,701 non-frail individuals with self-reported baseline (1998-2001) data available for the evaluation of energy-adjusted dietary inflammatory index (E-DIITM) (calculated from food frequency questionnaires), depressive symptoms (from the Center for Epidemiologic Studies Depression; CES-D), and follow-up frailty measurements (2011-2014). Frailty was defined as fulfilling ≥3 Fried frailty criteria. Results are presented by baseline CES-D scores <16 or ≥16 points, which denotes the absence or presence of clinically relevant depressive symptoms, respectively. Logistic regression estimated odds ratios (OR) and 95% confidence intervals (95% CI) between E-DII and frailty onset, adjusting for confounders. In all study participants, mean (standard deviation; SD) age was 58(8) years and E-DII was -1.95 (2.20; range: -6.71 to +5.40, higher scores denote a more pro-inflammatory diet), and 45% were male. In those without clinically relevant depressive symptoms, one-unit higher E-DII score was associated with 14% increased odds (95% CI:1.05-1.24) of frailty. In those with depressive symptoms, one-unit higher E-DII score was associated with 55% increased odds of frailty (95% CI: 1.13-2.13). The association between inflammatory diet and increased odds of frailty appeared somewhat stronger among those with depressive symptoms. This preliminary finding warrants further investigation.

